# CdSe/ZnS Quantum Dot-Labeled Lateral Flow Strips for Rapid and Quantitative Detection of Gastric Cancer Carbohydrate Antigen 72-4

**DOI:** 10.1186/s11671-016-1355-3

**Published:** 2016-03-11

**Authors:** Xinyu Yan, Kan Wang, Wenting Lu, Weijian Qin, Daxiang Cui, Jinghua He

**Affiliations:** Out-Patient Department, Zhujiang Hospital, Southern Medical University, 253 Gongye Road, 510280 Guangzhou, Guangdong People’s Republic of China; Institute of Nano Biomedical and Engineering, Department of Instrument Science and Engineering, School of Electronic Information and Electrical Engineering, Shanghai Jiao Tong University, 800 Dongchuan Road, 200240 Shanghai, People’s Republic of China; National Center for Translational Medicine, Collaborative Innovational Center for System Biology, Shanghai Jiao Tong University, 800 Dongchuan Road, 200240 Shanghai, People’s Republic of China

**Keywords:** Gastric cancer, CA72-4, CdSe/ZnS quantum dots, Lateral flow strip

## Abstract

Carbohydrate antigen 72-4 (CA72-4) is an important biomarker associated closely with diagnosis and prognosis of early gastric cancer. How to realize quick, sensitive, specific, and quantitative detection of CA72-4 in clinical specimens has become a great requirement. Herein, we reported a CdSe/ZnS quantum dot-labeled lateral flow test strip combined with a charge-coupled device (CCD)-based reader was developed for rapid, sensitive, and quantitative detection of CA72-4. Two mouse monoclonal antibodies (mAbs) against CA72-4 were employed. One of them was coated as a test line, while another mAb was labeled with quantum dots and coated onto conjugate pad. The goat anti-mouse IgG was immobilized as a control line. After sample was added, a sandwich structure was formed with CA72-4 and these two mAbs. The fluorescent signal from quantum dots (QD)-labeled mAb in sandwich structure was related to the amount of detected CA72-4. A CCD-based reader was used to realize quantitative detection of CA72-4. Results showed that developed QD-labeled lateral flow strips to detect CA72-4 biomarker with the sensitivity of 2 IU/mL and 10 min detection time. One hundred sera samples from clinical patients with gastric cancer and healthy people were used to confirm specificity of this strip method; results showed that established strip method own 100 % reproducibility and 100 % specificity compared with Roche electrochemiluminescence assay results. In conclusion, CdSe/ZnS quantum dot-labeled lateral flow strips for detection of CA72-4 could realize rapid, sensitive, and specific detection of clinical samples and could own great potential in clinical translation in near future.

## Background

Stomach cancer is the fourth most common cancer and the second leading cause of cancer-related death worldwide [1–3]. It ranks number two among all malignant tumors in China according to the latest cancer disease spectrum [4]. The gastric cancer prognosis is very poor with 5-year survivals below 24 % [5, 6]. Therefore, it is urgent to find an early diagnostic tool to increase the gastric cancer survival rate.

Up to date, clinical biomarkers for diagnosis of early gastric cancer are still very few. For example, so far carcinoembryonic antigen (CEA) is often used to screen out or diagnose early gastric cancer patients in the hospital [7, 8]; nevertheless, due to the low sensitivity and specificity, CEA examination cannot screen out most early gastric cancer patients, therefore, to look for new biomarkers associated with gastric cancer diagnosis is an important task. In recent years, gastric cancer-related carbohydrate antigen 72-4 (CA72-4) has been shown a higher sensitivity in early gastric cancer or recurrent cases than CEA, particularly the specificity of CA72-4 reached to 97 % in 58 disease-free objects (56 out of 58 were undetected) [9]. These reports highly suggested that CA72-4 may be considered as a specific biomarker of gastric cancer. In the different stages of gastric cancer, CA72-4 antigen was released into the blood circulation system and leaded to different levels in sera samples [10, 11]. Studies correlating levels of CA72-4 with findings of pathologic exams in gastric cancer have shown significantly higher levels associated with gastric serosal invasion caused by gastric cancer, lymph nodal metastases, and invasion of veins or lymphatic vessels into the gastric wall [12–15]. All these studies suggested that levels of CA72-4 in the sera could efficiently predict different stages of gastric cancer. Therefore, in this study, we selected CA72-4 antigen as our detection target molecule.

Immunochromatographic test strip (ICTS) has been widely used in qualitative and semi-quantitative detection of biomarkers. This technology uses antigen-antibody reaction features to detect numbers of analytes, including antigens, antibodies, and even the yields of nucleic acid amplification tests [16–19]. This technology has the advantages such as user-friendly format, rapid detection, long-term stability, and relatively low cost. Traditional colloidal gold lateral flow tests are analyzed by naked eyes, which is subjective and inaccurate. For these reasons, several groups developed electrochemical immunosensor and CdSe or CdTe quantum dot-labeled lateral flow strips combined with charge-coupled device (CCD)-based reader for ultrasensitive quantitative detection of antibody or antigen in Shanghai Jiao Tong University [20, 21]. Although CdTe and CdSe exhibited strong fluorescent signals, as the used time was extended for more than 6 months, the prepared quantum dot (QD)-labeled lateral flow strips displayed decreased fluorescent signals in the course of clinical sample examination; how to keep the stable fluorescent signals in prepared QD-labeled lateral flow strips has become a challenge.

In this study, CdSe/ZnS quantum dots were selected and labeled with anti-CA72-4 monoclonal antibody. A new type of stable flow strips were developed to quantify the CA72-4 antigen combined with the previous in-house-developed CCD-based reader. In order to exam the detection sensitivity, specificity, speed, and repeatability of the prepared lateral flow strips, 100 specimens were collected and results have been compared with the results of Roche electroluminescent assays.

## Methods

### Chemicals and Materials

**Fig. 1:**
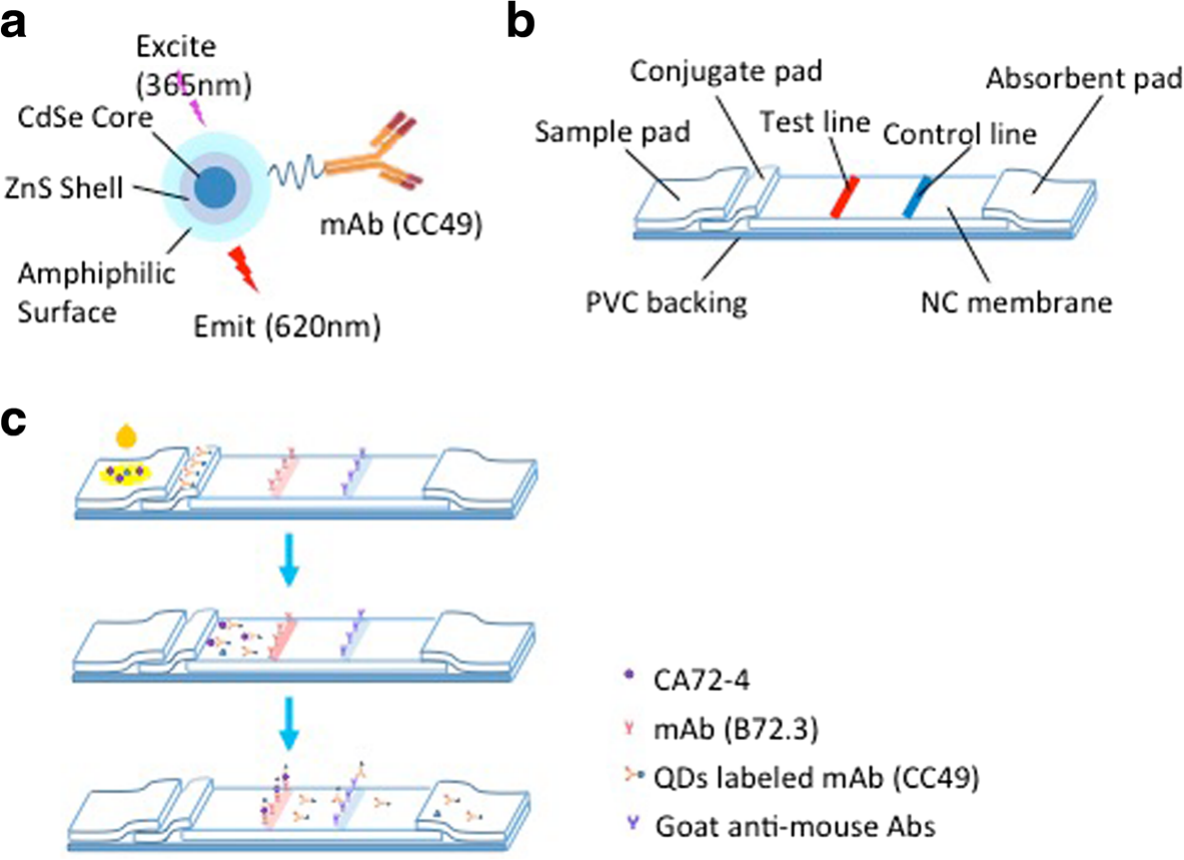
Schematic diagram of QD-based ICTS. **a** Conjugation of CdSe/ZnS QDs with CA72-4 mAb (CC49). **b** Structure of QD-based ICTS. **c** Sample containing CA72-4 was dropped to sample pad and migrated along the ICTS. Firstly, CA72-4 in the samples combined with QDs probeat conjugate pad. The formed complexes continued to migrate along the membrane and were captured by B72.3 on test line and formed QD-labeled CC49-CA72-4–B72.3 complexes. As the liquid sample continued migrating, the residual CC49 was captured by the goat anti-mouse IgG immobilized on control line. The excess QD conjugates continued to migrate towards the absorbent pad

Water-soluble CdSe/ZnS quantum dots (excitation and emission wavelengths were 365 and 620 nm, respectively) were obtained from Najing Technology Co. Ltd. (China) (Fig. 1a).The QDs were with carboxyl groups on their surface and were 5–7 nm in diameter. CA72-4 is commonly detected through monoclonal antibody CC49 and B72.3 [22–24]. B72.3 and CC49 were purchased from Shanghai Cnpair Biotech Co. Ltd. (China). Goat anti-mouse IgG polyclonal antibody and CA72-4 antigen were also provided by Shanghai Cnpair Biotech Co. Ltd. (China). Bovine serum albumin (BSA) was obtained from Sigma-Aldrich (USA). 1-Ethyl-3-(3-dimethylaminopropyl) carbodiimide hydrochloride (EDC), 2-(*n*-morpholino) ethanesulfonic acid (MES) and *n*-hydroxysuccinimide (NHS) were from Shanghai Aladdin Industrial Corporation (China). Other chemicals (analytical grade) were obtained from Sigma Company. PALL Vivid170 Nitrocellulose (NC), Bilbulous paper, semirigid polyvinylchloride (PVC) sheets, glass fiber using for conjugate pad, sample pad, and adsorbent pad were all supplied by Shanghai JieYi Biotechnology Co., Ltd. (China). Fluorescence spectrophotometer was purchased from Hitachi High-Technologies.

### Clinic Samples

This study was approved by the Medical Ethics Committee of Zhujiang Hospital. Informed consent was obtained from patients. Human experimentation guidelines of the Zhujiang Hospital were followed in the conduct of clinical research. In this study, we selected 70 CA72-4-positive serum samples from gastric cancer patients collected at the Out-Patient Department, Zhujiang Hospital, during October 2013 to August 2015. The negative control group consisted of 30 healthy CA 72-4-negative sera from volunteer blood donors. All samples were collected and kept at −70 °C refrigerator for further use.

### Preparation of CA72-4 mAb Labeled With CdSe/ZnS QDs

The CdSe/ZnS quantum dots were obtained from Najing Technology Co. Ltd. (China), and characterized by UNICAM UV 300 Spectrophotometer (Thermo Spectronic, USA), high-resolution transmission electron microscope (TEM), and PerkinElmer LS 55 Spectrofluorimeter. For conjugation of CA72-4 mAb with CdSe/ZnS quantum dots with carboxyl group (Fig. 1a), all steps were protected from light to avoid fluorescent quenching of QDs. In brief, QDs was sequentially pretreated with EDC and NHS. Then, QDs and EDC in 1:4000 (mol/mol) were added to 0.01 M MES (pH = 6.0) with continuous gentle vortex mixture for 15 min. NHS (QDs: NHS was 1:2000, mol/mol) was added after wash with 0.02 M PBS (pH = 7.2) and further incubated for 15 min. Next, the pretreated QDs were mixed with anti-CA72-4 mAb, CC49, in 1:8 (mol/mol), following with incubation in room temperature for 3 h and 17 °C overnight. Finally, QD-conjugated CC49 was blocked using 1.5 % BSA in PBS for 30 min after wash by centrifugation. The efficient of conjugation was checked though BCA protein assay kit (Beyotime Biotechnology, China) and running of 50 % agarose gel in 80 V, 220 mA for 40 min. In addition, the shape of QDs after conjugation was observed through TEM.

### Preparation of Lateral Flow Test Strips

The lateral flow test strip was composed of sample pad, conjugate pad, NC membrane, and absorbent pad, and all parts were pasted on a PVC baking (Fig. 1b). The sample pad was saturated with PBS containing 0.1 % Tween-20 (*v*/*v*) and dried at room temperature. The prepared QD-labeled anti-CA72-4 mAb (CC49) was applied into the conjugate pad, dried at 37 °C overnight and stored at 4 °C. To prepare the test line and control line, Biodot BJQ 3000 model XYZ 3200 was used. A solution of anti-CA72-4 mAb (B72.3, 2 mg/mL) was immobilized on NC membrane as a test line. The goat anti-mouse IgG was immobilized at a concentration of 1 mg/mL as a control line. After immobilization, the NC membrane was dried at 37 °C for 1 h and then blocked with PBS containing 1 % BSA (*w*/*v*). Absorbent pad was not treated. The sample pad, conjugate pad, coated membrane, and absorbent pad were sequentially laminated and pasted to a PVC backing pad with proper overlaps to ensure that the testing solution could migrate through the whole test strip. Then, the whole assembled strip was cut lengthways into strips with 3 mm width and stored in sealed plastic tubes until use.

### Selection of CCD-Based Lateral Test Strip Reader

The lateral test strip reader is a very key tool for quantitative detection of CA72-4. In previous reports, our group has developed a CCD-based reader to detect QD fluorescent signals quantitatively [21]. In this study, we chose this portable fluorescence immunoassay chip detector as the test strip reader. In addition, a UV laser was used to directly observe the fluorescence on the lateral flow test strips.

### Assay Procedure

To perform the fluorescence assay, the prepared QD-based ICTS was put on a clean horizontal platform, and a desired volume of the standard antigen CA72-4 was dropped into the sample pad and migrated towards the other end of the strip under capillary action (Fig. 1c). PBS without CA72-4 was used as control. Each sample test was repeated for three times under the same condition. After immune reaction for 10 min, the test strip was placed into the tests strip reader, followed by recording fluorescence intensity of test line and control line. Further, 70 serum samples from CA72-4-positive patients and 30 serum samples from CA72-4-negative healthy volunteers were detected. All the serum samples were determined with electrochemiluminescence assay kit of Roche.

### Data Analysis

All data are presented in this paper as means ± SD. Standard curve was produced from GraphPad Prism 5 (GraphPad Software, San Diego, USA). Data was also analyzed using GraphPad Prism 5; statistical differences were evaluated using the *t* test and considered significance at *P* < 0.05 level. Roche electroluminescent assay is considered as a golden standard method to detect gastric cancer CA72-4 antigen. *X*^2^ was employed to get sensitivity and specificity compared with the golden standard method.

## Results and Discussion

### Characterizations of QDs and Anti-CA72-4 mAb Labeled With QDs

**Fig. 2:**
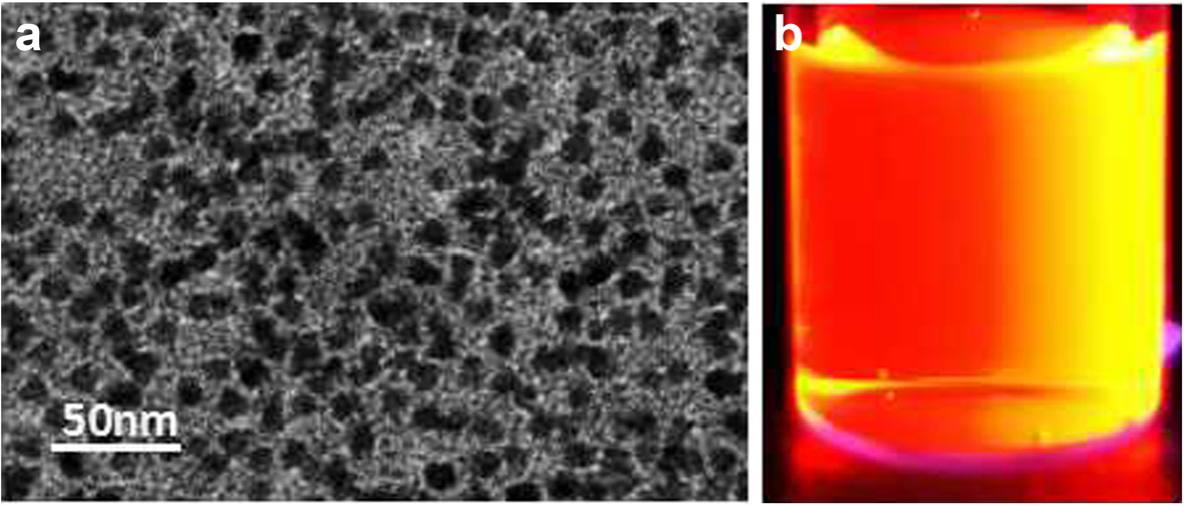
Characterizations of QDs. **a** TEM of QDs. **b** Exciting of QDs in UV light

**Fig. 3:**
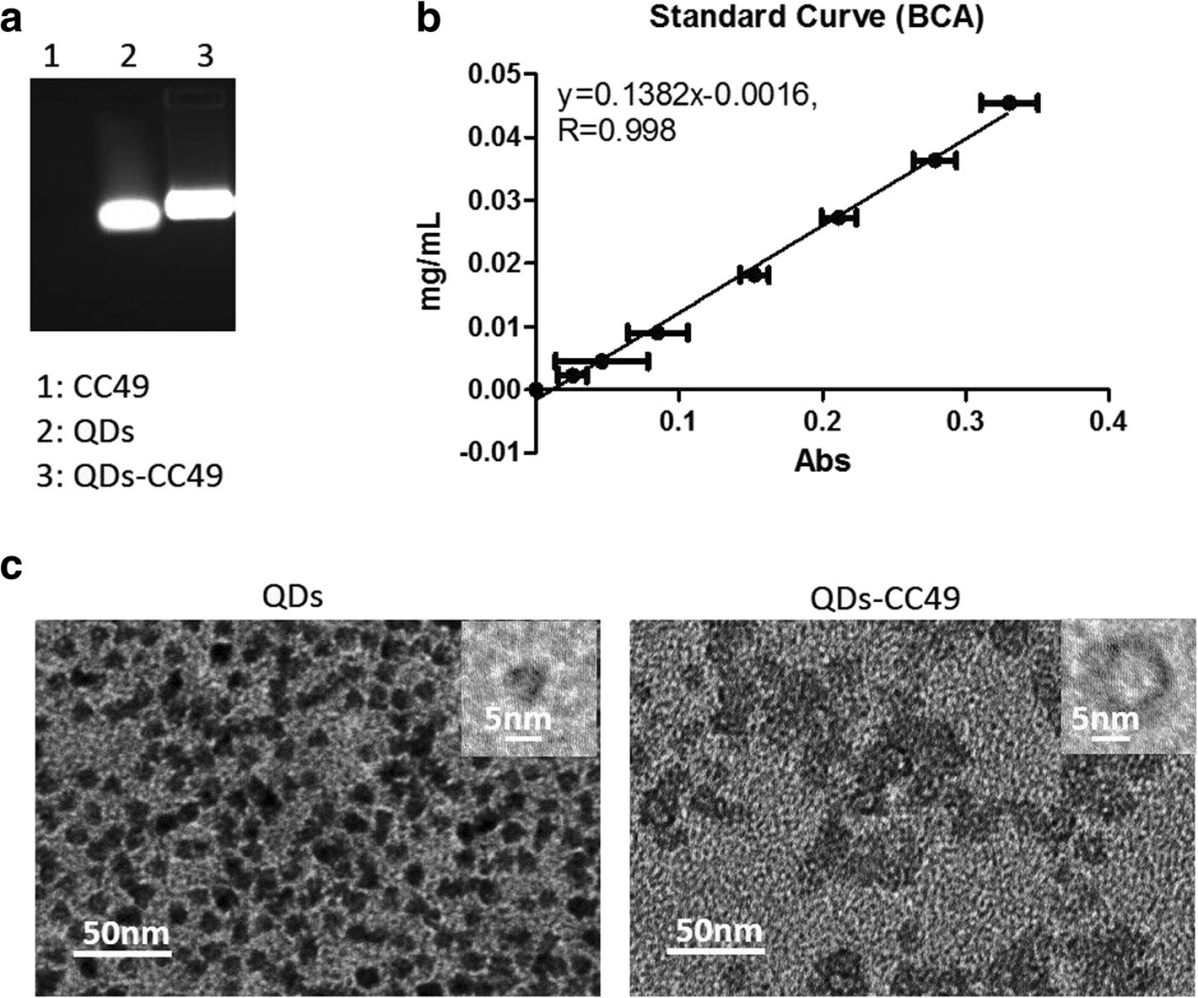
Conjugation of QDs with CC49. **a** Naked QDs and QD-labeled CC49 were run on a 50 % agarose gel. **b** Standard curve for detecting unlabeled CC49 in the supernatant of conjugation through a BCA protein assay kit. **c** TEM of naked QDs and QD-labeled CC49. The results of three independent experiments showing a similar trend are depicted

Water-soluble CdSe/ZnS quantum dots (excitation and emission wavelengths were 365 and 620 nm, respectively) were purchased from commercialization company. The QDs were modified with carboxyl groups on their surface and were 5–7 nm in diameter. The QDs had a good dispersion as shown in Fig. 2a. QDs were emitted under the laser at a wavelength of 620 nm. Under the 365-nm UV light excitation, the images in Fig. 2b showed the relative photo luminescence intensity and fluorescence image of QDs. To improve the sensitivity of detecting mAb, CC49 was conjugated with QDs as probes. To evaluate the efficiency of conjugation of QD-labeled CC49, QD-labeled CC49 were loaded in a 50 % agarose gel firstly. The different fluorescence bands were observed under the UV light after the electrophoresis. CC49 (160 kDa) labeled with QDs shows 890 kDa. The QD-labeled CC49 presents a larger molecular weight and ran slower than naked QDs (Fig. 3a). In order to anticipate the ration of conjugation, BCA protein assay kit was used to detect the unlabeled CC49 in the supernatants. After centrifugation, the QD-labeled CC49 was in the bottom of centrifugation tubes. A formula was generated using protein standards (*y* = 0.1382*x* − 0.0016, *R* = 0.998) (Fig. 3b). The unlabeled antibody in the supernatant, after separating QD-labeled CC49 with centrifugation, was collected. We then quantify both the total CC49 and unlabeled CC49 with BCA assay. The QD-labeled CC49 was calculated via the total antibody minus the unlabeled antibody. The ratio between QD-labeled CC49 and total CC49 has been counted as 17.85 ± 4.501 % (mean ± SD, *n* = 10). Since CC49 and QDs used for conjugation were 8:1 (mol/mol), the real conjugation efficiency of CC49 and QDs was dramatically high. We finally used TEM to observe the shape change after conjugating CC49 with QDs. We found that the QD-labeled CC49 formed loose and larger particles (Fig.3c, right), while naked QDs particles were smaller in size and deep in color (Fig. 3c, left). Taken together, an effective conjugation of QDs with anti-CA72-4 mAb was well performed in this study.

### Emission and Excitation of QDs Probe

**Fig. 4:**
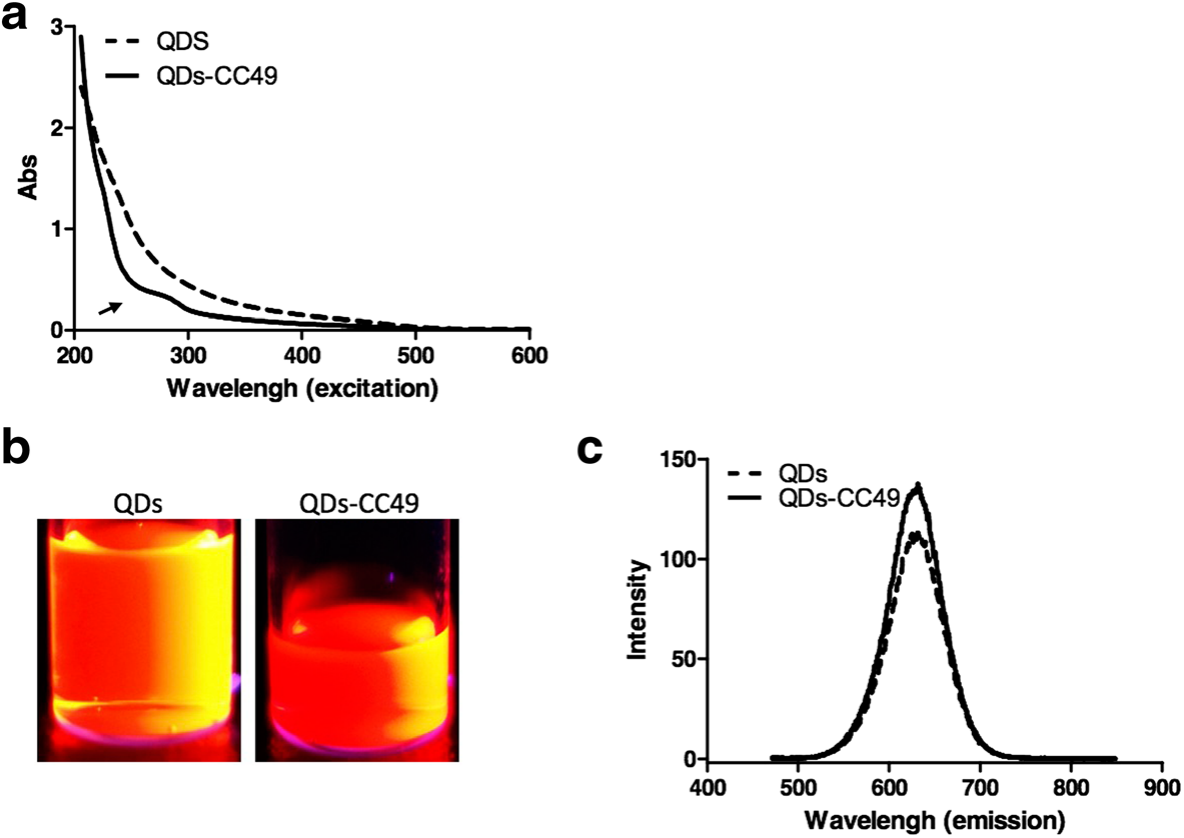
Emission and excitation of QD-labeled CC49. **a** Naked QDs and QD-labeled CC49 were excited from 200 to 400 nm. A distinctive small absorbance peak around 280 nm was observed on the QD-labeled CC49. **b** Naked QDs and QD-labeled CC49 were excited at 365 nm. Emission was observed with a UV laser. **c** The emission peaks of QDs and QD-labeled CC49 were formed at 620 nm. The results of three independent experiments showing a similar trend are depicted

Although we have gotten an efficient conjugation of QDs and CC49, an anti-CA72-4 mAb, whether the emission and excitation of QD-labeled CC49 were same as naked QDs remains unknown. Naked QDs and QD-labeled CC49 were excited at 365 nm. To determine the characterization of excitation of the QD-labeled CC49, we firstly used a continuous range of excitation light from 200 to 600 nm to check the characterization of excitation light for QD-labeled CC49. Absorbance of samples was detected. The QD-labeled CC49 showed a similar manner with naked QDs, which excited at 200 to 400 nm (Fig. 4a). We then observed the emission with a UV laser. Both QDs and QD-labeled CC49 showed bright emitted lights (Fig. 4b). Moreover, emitted lights were also measured with spectrophotometer. QD-labeled CC49 exhibited the strong fluorescence signal and narrow emission spectra as naked QDs, which presented an emission peak at 620 nm (Fig. 4c). Moreover, QD-labeled CC49 also showed a distinctive small absorbance peak around 280 nm, as a characterization of proteins, indicating that antibody was included. In all, our QD-labeled CC49 has the emitting and exciting features coming from both monoclonal antibody and QDs, suggesting this QD-labeled CC49 is appropriate for detection of CA72-4.

### Detection of CA72-4 Using QD-Based ICTS

**Fig. 5:**
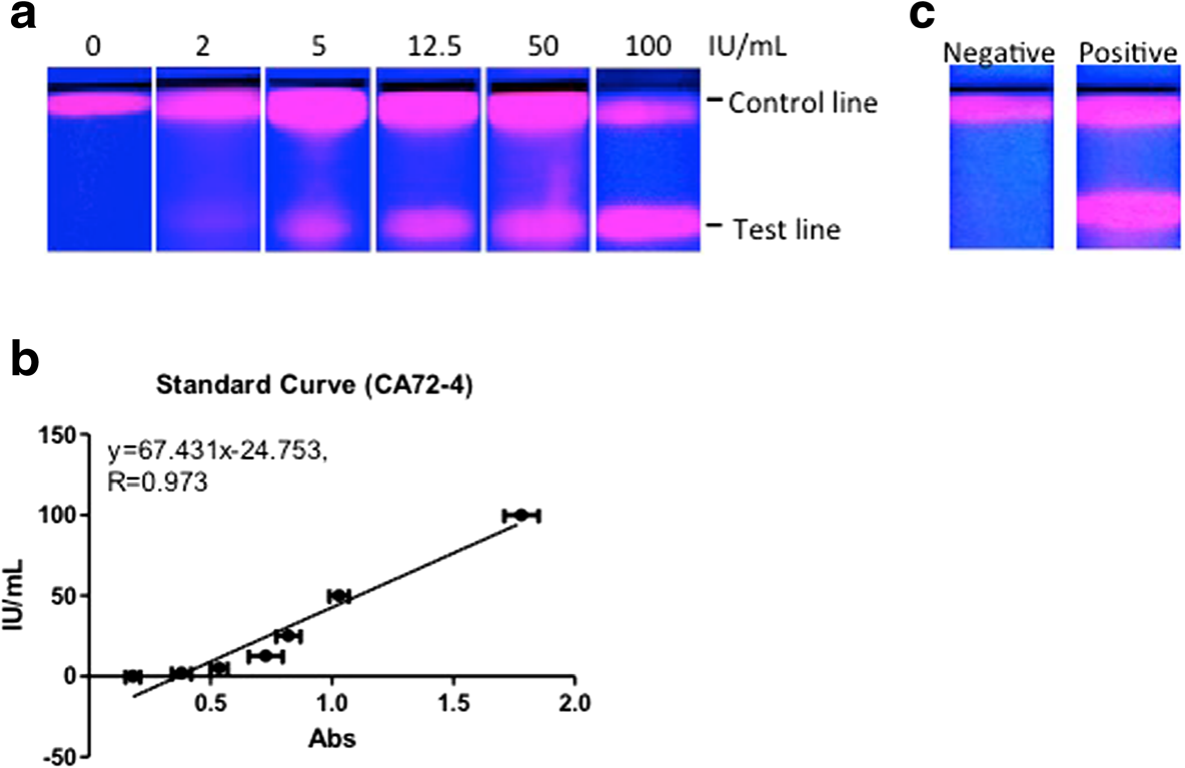
Detection of CA72-4 with QD-based ICTS. **a** Detection of purchased CA72-4 (5 IU/mL) by a UV laser. **b** Detection of CA72-4-negative and CA72-4-positive serum from patients by a UV laser. **c** Standard curve for detecting CA72-4 through data from a portable fluorescence immunoassay chip detector

As the ICTS was successfully fabricated through efficiently conjugating QDs and an anti-CA72-4 mAb, CC49, we proposed to use it to detect CA72-4. A purchased CA72-4 antigen was firstly employed. Samples were dropped to ICTS, and 10 min later, the fluorescence emitted from test line, coated CA72-4 mAb (B72.3), and control line, coated with goat anti-mouse IgG, on ICTS were observed in UV light. We found a dose-dependent increase of the intensity of fluorescence following with the concentrations of CA72-4 in series dilution (from PBS as control, 2, 5, 12.5, 25, 50, and 100 IU/mL), which also showed the lower limit to 5 IU/mL (Fig. 5a). To get a quantitative data of CA72-4, furthermore, the portable fluorescence immunoassay chip detector was used. Series diluted CA72-4 (from PBS as control, 2, 5, 12.5, 25, 50, and 100 IU/mL) were measured and provided a standard curve (*y* = 67.431*x* − 24.753, *R* = 0.973) (Fig. 5b). Notably, our QD-based ICTS showed extreme sensitivity by using the portable fluorescence immunoassay chip detector (lower limit reached to 2 IU/mL) (Fig. 5b).

We also kept and prepared ICTS in the 4 °C for 1 year and examined the fluorescent signals by using CCD-based reader one time per week. Results showed that no fluorescent signal intensity difference was detected during the period of 1 year (*P* > 0.05), which fully suggest that CdSe/ZnS quantum dot-labeled anti-CA72-4 mAb had stable fluorescent signal, and had good repeatability, was better than other QDs such as CdTe or CdSe QDs.

### Clinical Sample Test

The QD-based ICTS was used to test 100 clinical samples. Sera from CA72-4-negative or CA72-4-positive patients, which had been determined with electrochemiluminescence assay, were used to evaluate our QD-based ICTS. A dramatic fluorescent band in test line and control line were observed in the CA72-4-positive samples rather than in CA72-4-negative samples (Fig. 5c). Since, CA72-4, only higher than 6 IU/mL in the blood from patients, is suggested to be significance for diagnosis, our ICTS was capable for clinical application. Therefore, we used serum samples from 70 CA72-4-positive and 30 CA72-4-negative patients. The specificity and sensitivity of this QD-based ICTS were determined. The QD-based ICTS presented a perfect reproducibility compared with the assay results from Roche (Table 1). One hundred percent sensitivity and 100 % specificity were found in detection of patient and healthy volunteer serum samples. The developed QD-based ICTS own obvious advantages such as sensitive, rapid, specific, and quantitative assay.

**Table 1:** Detection of results of CA72-4 in serum samples using QD-based ICTS

Serum samples	ICTS			Validity (95 % CI)
	Positive	Negative	Total	
Roche assay positive (*n* = 70)	70	0	70	Sensitivity 100 % (91–100)
Roche assay negative (*n* = 30)	0	30	30	Sensitivity 100 % (91–100)

Regarding the diagnosis of gastric cancer, imaging, and endoscopic examinations usually play important roles [25, 26]. However, these examinations are complex and expensive. Detecting tumor biomarkers, such as CEA and CA72-4, has been considered as a rapid and simple method for screening and diagnosis of early gastric cancer [27]. CA72-4 has a higher sensitivity to distinguish gastric cancer patients and disease-free healthy population than other markers, such as CEA [9]. Therefore, in this work, ICTS based on QD-labeled mAb for CA72-4 detection were developed. The QD-based ICTS was not only rapid, low cost, and easy in handle but also extremely sensitive and quantitative. Nevertheless, it has also been suggested that the sensitivity is not entirely desirable in cancer detection by using a single marker [9]. Some researchers have shown that combining the CA72-4 with other markers, such as CEA, CA199, and CA125, could dramatically improve the sensitivity [28–30]. Our strategy also can be easily altered to detect these molecules. Anyhow, QD-based ICTS is a powerful tool in detection of targets and can be applied potentially in screening clinical gastric cancer patients.

## Conclusions

To develop a convenient and sensitive strategy for detection of CA72-4, the gastric cancer marker, in this study, we conjugated the anti-CA72-4 mAb with QDs, which provided a fluorescence signal for recognition. Then, we constructed an ICTS by using the QD-labeled mAb and another unlabeled mAb, which used for capture of CA72-4. This ICTS is simple in operation and gets sensitive quantitative results, which had reached to 2 IU/mL. One hundred percent sensitivity and 100 % specificity were found in detection of patients serum samples, comparing to the electrochemiluminescence assay from Roche. In our QD-based ICTS system, the same strategy can be used for development of strip for several markers, which may improve the sensitivity of diagnosis. It is the first time, to our knowledge, to use QD-based ICTS to detect CA72-4. This ICTS is potentially be used as a clinical diagnostic reagent for gastric cancer, which may help forecast tumor progression and benefit following therapy.

## Abbreviations

BSA: bovine serum albumin
CA72-4: carbohydrate antigen 72-4
CCD: charge-coupled device
CEA: carcinoembryonic antigen
ICTS: immunochromatographic test strip
NC: nitrocellulose
QDs: quantum dots
